# The barriers and facilitators to routine outcome measurement by allied health professionals in practice: a systematic review

**DOI:** 10.1186/1472-6963-12-96

**Published:** 2012-05-22

**Authors:** Edward AS Duncan, Jennifer Murray

**Affiliations:** 1 NMAHP Research Unit, Iris Murdoch Building, University of Stirling, Stirling, FK9 4LA, Scotland, UK

**Keywords:** Allied health professional, Routine outcome measurement, Outcome measurement, Facilitators, Barriers, Occupational therapy, Physical therapy, Physiotherapy, Speech and language therapy

## Abstract

**Background:**

Allied Health Professionals today are required, more than ever before, to demonstrate their impact. However, despite at least 20 years of expectation, many services fail to deliver routine outcome measurement in practice. This systematic review investigates what helps and hinders routine outcome measurement of allied health professionals practice.

**Methods:**

A systematic review protocol was developed comprising: a defined search strategy for PsycINFO, MEDLINE and CINHAL databases and inclusion criteria and systematic procedures for data extraction and quality appraisal. Studies were included if they were published in English and investigated facilitators and/or barriers to routine outcome measurement by allied health professionals. No restrictions were placed on publication type, design, country, or year of publication. Reference lists of included publications were searched to identify additional papers. Descriptive methods were used to synthesise the findings.

**Results:**

960 papers were retrieved; 15 met the inclusion criteria. Professional groups represented were Physiotherapy, Occupational Therapy, and Speech and Language Therapy. The included literature varied in quality and design. Facilitators and barriers to routine outcome measurement exist at individual, managerial and organisational levels. Key factors affecting professionals’ use of routine outcome measurement include: professionals’ level of knowledge and confidence about using outcome measures, and the degree of organisational and peer-support professionals received with a view to promoting their work in practice.

**Conclusions:**

Whilst the importance of routinely measuring outcomes within the allied health professions is well recognised, it has largely failed to be delivered in practice. Factors that influence clinicians’ ability and desire to undertake routine outcome measurement are bi-directional: they can act as either facilitators or barriers. Routine outcome measurement may only be deliverable if appropriate action is taken at individual therapist, team, and organisational levels of an organisation.

## Background

The current economically challenging climate of the developed world has seen significant restrictions being placed on professionals and services [1], the requirement for clinicians to demonstrate the impact of their practice has never been more vital [2]. One way to achieve this is through the routine measurement of patient outcomes. Outcome measures are assessments that measure change in patients functioning, performance or participation over time. Routine outcome measurement is strongly mandated for in national health policy [3,4]. However, despite continued professional appeals [5,6] this standard has remained largely aspirational with little evidence of routine outcome measurement in allied health professional practice, and continued evidence of ambivalence towards outcome measurement by staff [7-11]. Whilst this position has never been professionally justifiable, it existed and was, arguably, tolerated in a period of economic growth and clinical service expansion in the NHS (UK) and beyond. These times have past, and without strong evidence of impact allied health professional services are vulnerable to closure [1]. Routine outcome measurement is now, therefore, essential, as it is through such measurement that service impact can be evidenced.

There are several reasons to promote the use of routine outcome measurement in practice. Without routine outcome measurement clinicians receive little feedback on the types of outcomes that they achieve and on how these outcomes compare with other health professionals [12]. Records of patient outcomes also enables progress, which can sometimes appear intangible, to be effectively communicated to patients; and also promotes efficient treatment planning [12,13]. Routine outcome measurement can also be used to support the clinical justification of interventions, and provide important supporting evidence to healthcare funding bodies [2].

Despite these reasons, routine outcome measurement has largely failed to become embedded in practice [7,9,11]. Trauer and colleagues [14] gave an indication of why this may be when they highlighted that resistance is a common reaction to innovation and change in health services’ routine practice. But after 20 years of trying to embed routine outcome measurement into the practice of allied health professionals, the argument that it is due to change and innovation alone is insufficient. It is necessary, therefore, to examine why it continues to prove such a challenging issue.

Some research into the barriers and facilitators of routine outcome measurement has already been conducted. In an unstructured review of the literature [15], cost, practicality, clinical relevance and a lack of knowledge over which outcome measures to choose were highlighted as potential barriers to their routine use in practice. Potential facilitators were highlighted as ease of administration, speed of administration, measures that were easy to score and provided useful clinical information. In a more rigorous review in mental health services [14], staff concerns relating to routine outcome measurement were grouped into five domains: access to appropriate technology and ability to use it; appropriateness of instruments; time burden; suspicion of management or government motives; and competence and confidence in using outcome data. Patterns of issues are evident across both these papers and it is intuitive that some of these issues may also be relevant to the allied health professions. Yet, other unidentified issues may also play an important role. A comprehensive review of the literature pertaining to routine outcome measurement in the allied health professions is therefore warranted.

## Methods

This review aimed to address the question: what are the barriers and facilitators to routine outcome measurement by allied health professionals in practice? Few studies define what they mean by routine outcome measurement in practice. This study adopts Colquhoun and colleagues’ [7] recent definition of routine outcome measurement as: “the systematic use of a standardised outcome measure(s) in clinical practice with every patient as a part of a standardised assessment practice guideline” (p.49). Outcome measures can be completed by either the patient or a therapist. This study includes both. A systematic literature review was conducted using an explicit search strategy to retrieve relevant publications. The review’s methods, search strategy and inclusion criteria used to identify relevant papers conform to established systematic review procedures [16]. No restrictions on professional group were applied at this stage in order to maximise the search’s sensitivity. As the identified literature was heterogeneous, a modified narrative synthesis [17] framework for mixed-methods reviews was applied during the quality appraisal, data extraction, analysis, and synthesis stages. The search strategy involved electronic searches of the electronic bibliographic databases MEDLINE (1966–2010), PsycINFO (1967–2010), and CINHAL (1982–2010) for published work. The search strategy comprised of two search filters: ‘outcome measures’ and ‘facilitators and barriers’. The ‘outcome measures’ search filter was adapted from the published search strategy of Gilbody, House and Sheldon (pp.91-96) [18] who investigated outcome measurement in psychiatric research and practice. The ‘facilitators and barriers’ search filter was developed in a series of iterations by both authors. The search strategy filters comprised relevant terms and synonyms combined with the BOOLEAN operator “OR” and were then combined using the BOOLEAN operator “AND”. Detailed information on the search terms can be consulted in Additional file 1. Additional papers were sought by hand searching the reference lists of papers which were included in the review. Retrieved papers were included if a) they were concerned with identifying or researching factors which acted as facilitators and/or barriers in the routine use of outcome measures by allied health professionals in practice; and b) were published in English. No restrictions on year of publication, type of outcome measurement, study design or publication type were applied. Papers were excluded if a) the topic covered was not of direct relevance (e.g., validating or standardizing an outcome measure, whereby the perceived facilitators would be largely theoretical and applicable to the trial of the particular measure alone); b) the sample was not clearly defined (e.g., where only a general term such as ‘clinicians’ was used); or c) if the sample was not composed wholly of allied health professionals, those being: arts therapists, chiropodists, podiatrists, dietitians, occupational therapists, orthoptists, physiotherapists, prosthetists, orthotists, radiographers, speech and language therapists.

Included papers were first categorised into six mutually exclusive domains [19]: quantitative research; qualitative research; mixed methods research; conceptual paper; opinion or literature review; practice based project or audit. Within each of these categories, the quality of each paper was assessed by one of the authors using a descriptive checklist based on the Centre for Reviews Dissemination and Research [16]. Quality appraisal was checked and confirmed by the other author and any differences were resolved following discussion.

Following published thematic analysis guidelines [20] and narrative analysis guidance [17], key factors were identified and extracted from each paper into a summary table by one of the authors (JM). Factors were then compared with each other to identify higher level themes. Themes were composed of factors that had occurred in several papers and/or mirrored themes already found in the general literature about barriers and facilitators to ROM. Themes were refined and synthesized through critical discussion between the authors until an agreement on the final themes was consensually reached.

Paper inclusion, quality appraisal, data extraction and data synthesis were undertaken by one of the authors (JM). JM identified 14 papers (of the final 15 included) to be included in the review. Of these 14, five were randomly selected [7,21-24] to blindly assessed for match on emerging themes by the other author (ED). Agreement was 100%. Of the remaining papers (not included in the review), a sample of 11 were blindly assessed for inclusion/exclusion. Of these, six were elected for possible inclusion by JM based on sample or possible suitability to the review’s aims. The other five papers were selected at random. Of the five random papers, agreement for exclusion from the review was 100% between the authors. Of the six papers selected for possible inclusion, following discussion, both authors agreed that one of the papers [9] fit the review’s requirements for inclusion. While agreements on papers were clearly high, any disagreements were resolved through discussion and clarification.

## Results

### Volume, nature and frequency

960 titles and abstracts were retrieved from the electronic database searches, and screened for inclusion. Of these, 54 papers were judged to be potentially relevant to the current review’s criteria and were subjected to full review. Twelve of these were deemed relevant to the inclusion criteria. A list of papers excluded from the review at this stage can be found in Additional file 2.

**Figure 1:**
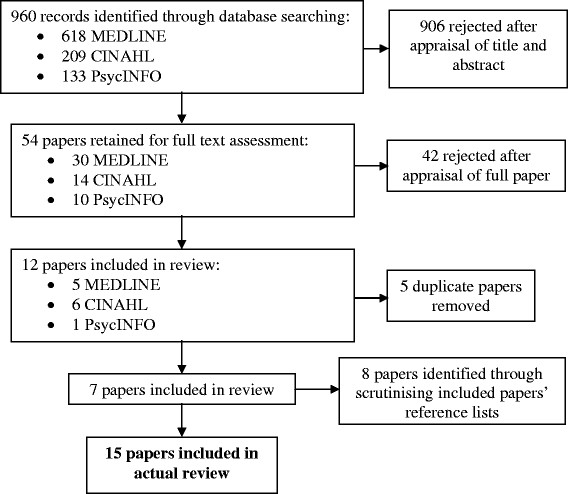
Process of paper identification and selection for review.

Five duplicate papers were removed and the remaining papers’ reference lists were subjected to scrutiny for sources that may have been missed in the original searches. Eight additional papers were identified following this reference list scrutiny and a total of 15 papers were retained for review (Figure 1).

**Figure 2:**
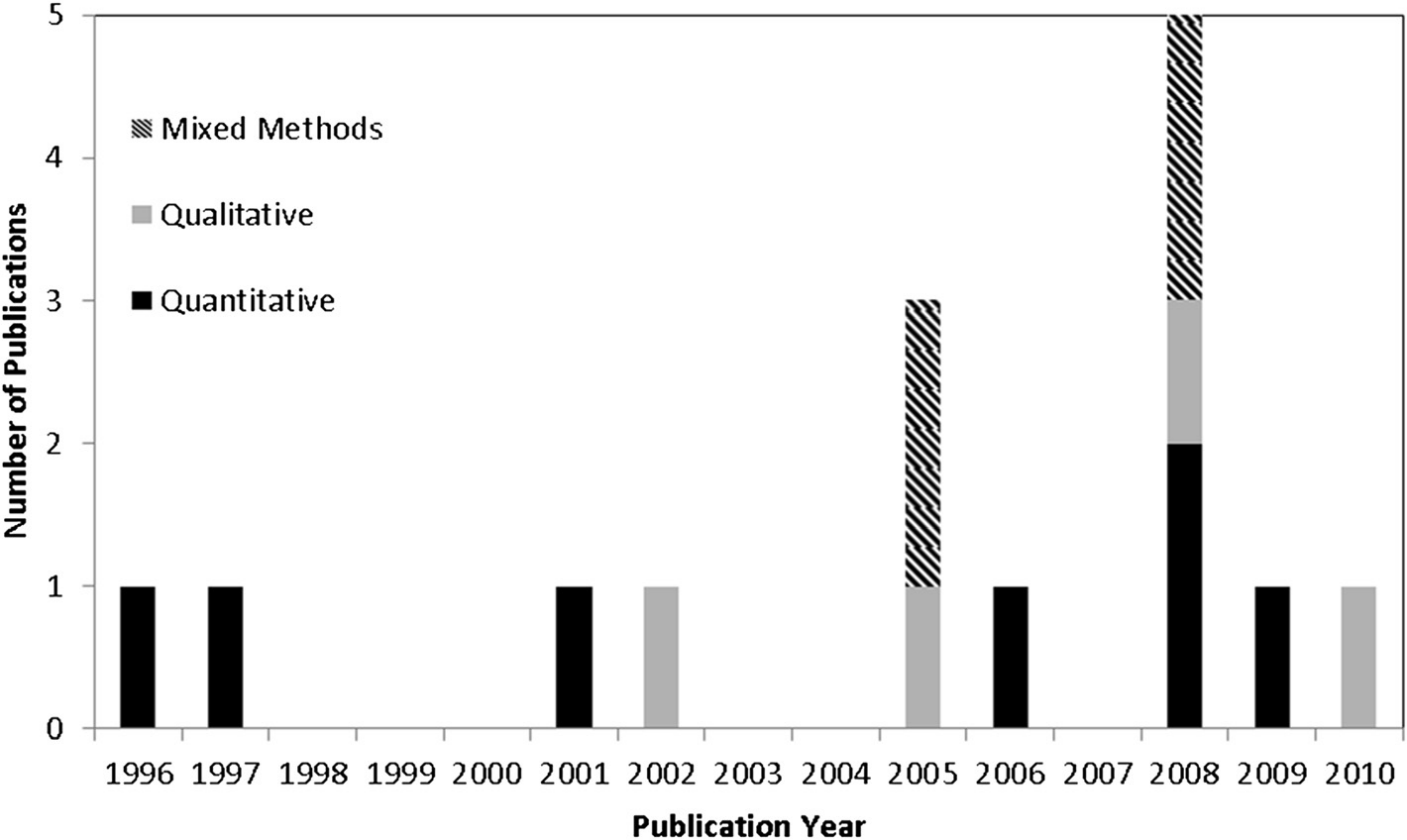
Number of included papers categorised by publication year and paper classification.

All of the included papers were research based, with eight quantitative in design, four qualitative and three using mixed methods. Three of the studies were conducted in the UK and Ireland, three in Canada, two in the US, one with a mixed sample from the US and Canada, two in Australia, two in New Zealand, one in Israel, and one in the Netherlands. A higher number of papers were published in 2008 (n = 5; 33%) than in any other year (Figure 2). No link to country of origin was present in relation to this spike as all five of these studies were conducted in different countries and across continents.

### Scope and quality

Of the 15 included papers, nine used a sample of physiotherapists, two used a mixed sample of physiotherapists and occupational therapists, two used a sample of occupational therapists, one used a sample of speech and language therapists, and one used a mixed sample of occupational therapists and speech and language therapists. Five papers investigated allied health professional managers or directors, seven investigated a general staff sample and two investigated a mixed manager/staff sample; one paper did not define whether their sample was staff or managerial. In total, 2161 allied health professionals participated in the reviewed studies, with the majority (N = 1450) being physiotherapists; see Additional file 3. Based on the quality appraisal, the papers were generally found to be adequately conducted, but several papers had various limitations (see Additional file 3). In general, the qualitative and mixed-methods papers’ were found to be more methodologically rigorous. Quantitative papers were of mixed quality. Several of the quantitative papers were unclear about their sampling in terms of sample size justification [21,25-28]; power analyses were only reported in one of the quantitative papers [17]. Many of the quantitative papers used simple descriptive analyses.

### Themes and concepts identified

A range of barriers and facilitators to routine outcome measurement by allied health professionals in practice were identified. The majority of the papers included in this review identified barriers to routine outcome measurement. Only one of the papers [21] focused their wording positively and consequently received more detail about facilitators. Five papers [21,22,29-31] reported facilitators and barriers as a continuum. Four higher level themes were identified: Knowledge, Education, and Perceived Value in Outcome Measurement; Support/Priority for Outcome Measure Use; Practical Considerations; Patient Considerations.

#### Knowledge, education, and perceived value in outcome measurement

This theme is composed of factors relating to outcome measure use at the individual clinician level. Eleven papers identified issues relating to clinicians’ knowledge as influencing routine outcome measurement usage. Eight papers [22,23,25-27,30,32,33] identified clinicians’ lack of knowledge about outcome measures’ reliability and validity as barriers to their use; whilst three papers [21,29,31] suggested that greater knowledge, understanding and familiarity of outcome measures’ increased the likelihood that they would be used in practice. One paper found that the use of outcome measures’ was more positively viewed by those with a Masters level qualification [12] and another identified that those who had a clinical specialty, as opposed to those who did not, were twice as likely to use outcome measures’ in practice [22]. The level of clinicians’ perceived value of outcome measurement use was discussed in seven papers [21,22,26,29-32]. Four of them recognised that this factor was bi-directional [21,29-31]. A lack of perceived value of outcome measures lead to a decreased likelihood of their use in practice, whilst greater perceived value appears to increase uptake.

#### Support and priority for outcome measure use

This theme relates predominantly to the influence of organisational factors on routine outcome measurement in practice. Low organisational priority and support for outcome measurement were identified as barriers in six papers [22,24,26-28,32]. Two papers indicated that having a high level of organisational commitment and support for routine outcome measurement facilitated their use [23,32]. Co-operation of colleagues [21] and the support of management [32] were also recognised as facilitating routine outcome measurement. Concerns about a lack of management support [32], inappropriate use of outcome data by managers to reproach staff [21,32], and the imposition of measurement tools by management were all cited as barriers to their use in practice [30]. At an individual level, clinicians appear to be more positive about outcome measurement when they have a choice over the selection of outcome measures which they consider to be the most useful or meaningful to their practice [23]_._

#### Practical considerations

This theme relates to practical issues and considerations relating to the use of routine outcome measurement in practice. Time was identified as an important influencing factor in ten papers. The barriers associated with lack of time involved not only the amount of time required for both patients and clinicians to complete an outcome measure [7,22,25,28], but also the number of patients seen by a clinician [24,31] and institutional restrictions which may limit the amount of time available to spend with patients [24]. Time was not considered in isolation, but in association with the clinicians’ assessments of suitability of a measure to their required context and the number of measures required [21,29,31]. Eleven papers [7,9,22-28,30,33] identified a lack of appropriate or available outcome measures as a barrier to their use. An outcome measure that was appropriate to the context, could be practically applied and did not require too much time to document [31] was recognised as increasing the chances of being used in practice [30,31]. Lack of funding or excessive costs of outcome measures [21,22,24,28-30] were clearly recognised as being barrier to their use.

#### Patient considerations

This theme relates to clinicians’ concerns about using outcome measures with and for their patients. The relevance of outcome measures to practice is of clear concern to clinicians. Six papers discussed how outcome measures which did not inform their practice were a barrier to their use [7,22,23,30,32,33]. Clinicians reported that information provided by outcome measures were too subjective or not useful to their practice [30,31,33], and that they do not help to inform or direct patient care [22,32]. Conversely, the opinion that outcome measures could support patients’ understanding, facilitate discharge planning, communication and treatment management [32], and the opinion that they provide the ability to make comparative clinical assessments [21] were likely to increase their use. This ‘fit’ of outcome measurement to routine practice was highlighted in five papers [7,21,24,29,32]. Three [21,24,32] identified that when there is a poor ‘fit’, barriers arose at both individual and organisational levels.

Two papers highlighted clinicians’ philosophical concerns about the relevance of standardised outcomes [30,31]; such concerns, however, were not found to be statistically related to outcome measure use [22]. Five papers reported clinicians’ concerns about their patients’ ability to complete outcome measures [21,22,24,30,33]. These included the belief that measures: could be too complicated to be completed independently [22,24]; were confusing [22]; required a reading level was too high [22]; presented language barriers for patients not fluent in English [22]; presented ethnic and cultural sensitivity issues [22,30]; and that patients may become disheartened if they viewed their progress as slow based on the outcome measurement findings [22]. Such perceptions were reported to decrease clinicians’ likelihood of using outcome measures in practice. Two papers, however, reported facilitating factors relating to patient considerations. Routine outcome measurement was viewed more favourably if they were easy for a patient to understand [22] and if patients did not find the measure to be too time consuming [21].

## Discussion

Routine outcome measurement has been strongly mandated for within the allied health professions for at least the last twenty years [34]. Whilst in times of economic growth and service expansion such measurement was generally perceived as optional, this is no longer the case. Embedding outcome measurement into routine practice is now essential in order to meaningfully communicate patient progress, promote efficient treatment planning, and demonstrate service impact and efficiency. This review investigated research examining the facilitators and barriers to routine outcome measurement in practice. The 15 included papers identified key factors in the literature. Information about these factors was systematically extracted, analysed and synthesised. The quality of the papers included in this review was mixed. Future papers in the field should be clearer about their analysis to aid readers’ understanding and interpretation. Despite this several different issues relating to the uptake of routine outcome measurement in practice were highlighted.

### Achieving routine outcome measurement in practice

Achieving routine outcome measurement in practice is challenging. As the collection of data usually occurs at the level of individual clinicians, it is natural to assume that this is where the ‘fault’ lies when routine outcome measurement is not achieved. The findings of this review suggest that this assumption should be questioned. There appear to be multi-level determinants to ensuring successful routine outcome measurement in practice [21,23,30,32]. Action is therefore required by organisations, teams and individuals if routine outcome measurement is to be achieved.

Organisations can increase the likelihood of successful routine outcome measurement by providing appropriate training, sufficient administrative support and adequate allocation of resources. Wherever possible the choice of outcome measures should not be organisationally imposed: external imposition of measures may inhibit their uptake [30]. This, however, is not always possible. Organisationally imposed outcome measures enable benchmarking both within and across services. Where external imposition of outcome measures does occur, organisations should consider developing mechanisms to overcome foreseeable barriers such as increasing communication to explain the rationale for compulsive measurement and increased education and training to counter the foreseeable resistance they will meet. Finally, organisations should carefully consider how they deal with sub-standard performance: a punitive approach to poor outcomes is likely to result in decreased measurement, not increased performance [21,32].

Teams should give priority to outcome measurement in practice. Sufficient time should be allocated to enable outcome measurement to occur. Several barriers were noted in the findings about the practical and patient concerns participants had relating to outcome measurement in practice [7,21,22,24,25,28,30,33]. Many of these could be addressed at a team level through a positive team culture and ethos of evaluation.

Clinicians too must take personal responsibility to ensure that they collect data to help them evaluate patient’s progress and their practice. The review highlighted two factors that appear to differentiate clinicians in respect of their attitude towards outcome measurement: those who worked in a clinical speciality and Higher Degree education [22,30]. It may be that there is a relationship between these factors that differentiate these individuals from others within their professional group. But both present challenges in the endeavour to achieve routine outcome measurement: personal interest cannot be imposed; and Higher Degree education is not currently mandatory within the allied health professions in the UK. However, for some professions this is the case elsewhere [35]. If the organisational and the team levels are supportive of routine outcome measurement in practice, then research in related fields [36,37] provides good empirical and theoretical reason to believe that the resultant social normative pressure will result in individual clinicians becoming more interested in collecting this data too. Knowledge of the clinician level factors that influence routine outcome measurement data collection may also be useful for managers when considering the profile of staff they wish to employ.

### Strengths and limitations of the review

This is the first time that a study has been undertaken to synthesise the key issues affecting routine outcome measurement exclusively in the Allied Health Professions. Systematic, transparent and rigorous methods were employed to gather and synthesise the available research and suggest areas of further investigation. The findings, however, must be interpreted with some caution. The databases that were selected were done on the basis of their quality and potential for indexing relevant journals. Further databases could have been included, but we were required to balance our desire for a comprehensive review with the high number of studies retrieved and the diminishing returns gained from searching multiple databases with high degrees of duplication. The considerable duplication in studies retrieved between databases gives a degree of confidence that the main papers that are indexed have been included within this review. There is a risk of bias arising from the fact that only English language articles were included and conferences proceedings and the grey literature were not searched. A randomly selected proportion of papers, double-coded to check for accuracy of paper inclusion, showed perfect agreement between the authors. Whilst this indicates that the inclusion criteria and paper section process was being closely adhered to, it is impossible to be absolutely certain that no errors were made at this stage. The quality of the included papers was mixed. Some papers were unclear about their sampling and power analyses were rarely reported; more detailed statistical analyses would have enabled a greater in-depth understanding.

In addition, only three different groups of allied health professionals were represented: physiotherapists, occupational therapists and speech and language therapists. The included studies reported both clinician and managerial perceptions. No attempt was made to categorise facilitators and barriers according to differing staff groups.

While a systematic process of searching for papers was followed, only seven of the 15 studies were identified via the search strategy, with the remaining eight being found via hand searching of reference lists. Developing the search strategy was a complex task that required considerable piloting to balance search sensitivity (ensuring that all relevant papers were being retrieved) with search specificity (ensuring that the papers that were retrieved were relevant). During the development of the search strategy, the various terms used to describe outcome measurement (e.g., outcome measurement, outcomes, standardised assessment, outcomes assessment) were considered, and a very broad search strategy was initially piloted. However, this returned more than 10,000 potential papers. The first ten returned pages of each of the three electronic databases were assessed and the fit of the papers based on title review were extremely poor: only one paper in a list of 30 returned pages was a possible fit. To refine the outcome measure search filter published search strategies on outcome measurement, in high quality publications, were then considered. Gilbody *et al.’s*[18] published search strategy on outcome measures was chosen as it appeared to fit extremely well with the aims of the current review. Their search strategy was then adapted to fit with the broader range of health profession groups that were targeted in the current review. The facilitators and barriers search filter was developed through a series of iterations and remained very broad, to capture as many prospective papers as possible. After examining the titles of the 15 included papers in the current review, we acknowledge that it may be the case that the outcome measure search filter could have been broader still, as numerous titles within the papers found via hand searching either did not contain any reference to outcome measurement (thus would logically have been missed in a broader search too) or used a much more generic term (e.g., standardised assessment) [8,25,29,33]. We did not include a search filter for standardised assessment as this has a very wide usage in many disciplines, from outcome measurement in health care to testing genetic disorders to personality assessment. Inclusion of standardised assessment as a search term would have resulted in an unmanageable number of paper returns, and the number of new, relevant papers would have been proportionately low. Through our additional hand searching of reference lists we feel that we have identified the relevant papers in the field that are currently published that our original searches missed. We therefore feel that while the sensitivity of the search strategy may have been an issue, the relevant papers to the review’s aims have been identified sufficiently. Finally, this review drew no data from grey material or conference proceedings. It is certainly possible that further data could be present in these sources and there is the potential for publication bias in our findings; however it was necessary to draw some boundaries around the inclusion criteria for the study and as a minimum quality standard we decided to only include papers that had been subject to peer review.

While the aim of the study was to investigate the facilitators and barriers to the routine use of outcome measures in practice, it is clear that many of these factors are bi-directional and can be viewed as either a barrier or facilitator depending on the emphasis given. With one exception^14^, the literature included within the review overwhelmingly sought to uncover the barriers to routine outcome measurement; considerably less emphasis was placed on discovering the facilitators. This may have led to important issues remaining undiscovered.

### Further research

While the current review’s findings appear relevant to allied health professional groups other than those included in the review findings, Wylie and Gallagher’s [38] study of transformational leadership identified that some of these groups are more inclined towards embracing change and modernisation than others: procedurally driven groups (e.g., radiographers and podiatrists; professions where procedures are often ‘medical’ in nature) are less likely to demonstrate transformation behaviours (e.g., motivation, intellectual stimulation, individualised consideration and behavioural and perceived charisma) than the more therapeutically focused groups (e.g., occupational therapists, physiotherapists). None of the former groups were included in this study’s findings. Further research is therefore warranted to investigate what, if any, impact these issues have on routine outcome measurement in practice.

This review gave no consideration to the environment in which data was being collected. Investigations of possible differences to routine outcome measurement in inpatient, outpatient, and domiciliary environments would be informative; whilst a number of key facilitators and barriers are shared; it is likely that there will also be variations between settings [21].

Almost all of the research to date has investigated staff perceptions of the barriers and facilitators of outcome measurement in practice. Only one paper [30] was included in this review which investigated whether the reported factors were associated with actual behaviours. It found that the perception that clinicians viewed patients as individuals and did not wish to ‘group’ or ‘categorise’ them through the use of outcome measurement was not statistically associated use of outcome measures in practice [30]. Clearly the association between the other factors reported in this review and actual behaviour should be studied in greater depth. This will enable both researchers and practitioners to identify which factors have greatest weight in predicting routine outcome measurement behaviours; which in turn will facilitate the development of interventions with a good theoretical and growing empirical basis to support routine outcome measurement in practice.

## Conclusions

The current review identified 15 studies that discuss the barriers and facilitators to routine outcome measurement by allied health professionals in practice. To date, the reasons for a lack of routine outcome measurement in allied health professionals’ practice have been focused at the level of individual clinicians. The findings of this review highlight that such an approach is likely to be insufficient: multi-level determinants may impact on the success or failure of routine outcome measurement in practice. There is an urgent need to investigate the association between the multi-level factors reported in this review and actual behaviour. This would enable the development and testing of theoretically driven evidence-based interventions to improve routine outcome measurement related behaviours in practice.

## Competing interests

This study was funded by a grant from the Scottish Government Health and Social Care Directorates. The funder did not have any involvement in the planning, execution, drafting or writing of the manuscript.

## Authors’ contributions

ED conceived of the review and led the development of the study protocol. JM conducted the literature search and data extraction. ED and JM undertook quality appraisal, analysis and synthesis. JM produced the initial draft manuscript. ED critically revised the manuscript for important intellectual content. ED and JM produced the final review. Both authors read and approved the final manuscript.

## Pre-publication history

The pre-publication history for this paper can be accessed here:

http://www.biomedcentral.com/1472-6963/12/96/prepub

## Supplementary Material

Additional file 1 Search strategy terms. The search terms used in each of the databases searched in the current review, and the number of articles returned at each stage. Click here for file

Additional file 2 List of studies excluded from the review following full paper appraisal stage (N = 42). A table containing the studies excluded from the current review following full paper appraisal. Click here for file

Additional file 3 Summary of quality appraisal information for included studies. A table containing the quality appraisal and study details for the 15 papers included in the current review. Click here for file
