# Supplementary material for: The barriers and facilitators to routine outcome measurement by allied health professionals in practice: a systematic review
Source: BMC Health Serv Res. 2012 May 22;12:96. doi: 10.1186/1472-6963-12-96 (PMC3358245; doi:10.1186/1472-6963-12-96)
Supplement: Additional file 1 — Search strategy terms. The search terms used in each of the databases searched in the current review, and the number of articles returned at each stage. [file 1472-6963-12-96-S1.doc]

The Barriers and Facilitators to Routine Outcome Measurement by Allied Health Professionals: A Systematic Review.

Additional File 1: Search strategy terms.

**MEDLINE (via PubMed): (hits)**

1. health status indicators 246

2. outcome and process assessment 3

3. outcome assessment 1533

4. quality of life 93798

5. outcome measure* 98561

6. health outcome* 11733

7. measure* 1715792

8. assess* 1244968

9. score* OR scoring 361880

10. index 325851

11. indices 82173

12. scale* 301743

13. monitor* 392414

14. #7 OR #8 OR #9 OR #10 OR #11 OR #12 OR #13 3333416

15. outcome* 596310

16. #14 AND #15 317624

17. # 1 OR #2 OR #3 OR #4 OR #5 OR #6 198176

18. #16 OR #17 397015

19. routine* 194229

20. #18 AND #19 14524

21. facilitat* 207729

22. barrier* 111795

23. #21 OR #22 326658

24. #20 AND #23 **618**

Title Sift 1 - 81

Abstract Sift 2 - 30

Full Paper Sift 3 - 5

**PsycINFO (via EBSCOhost): (hits)**

1. health status indicators 49

2. outcome and process assessment 15

3. outcome assessment 743

4. quality of life 24197

5. outcome measure* (apply related words) 15641

6. health outcome* (apply related words) 5238

7. measure* (apply related words) 373849

8. assess* (apply related words) 340910

9. score* OR scoring 184127

10. index 61225

11. indices 15964

12. scale* (apply related words) 200593

13. monitor* (apply related words) 40381

14. S7 OR S8 OR S9 OR S10 OR S11 OR S12 OR S13 814272

15. outcome* (apply related words) 145799

16. S14 AND S15 74686

17. S1 OR S2 OR S3 OR S4 OR S5 OR S6 43771

18. S16 OR S17 96771

19. routine* (apply related words) 19727

20. S18 AND S19 2025

21. facilitat* (apply related words) 72072

22. barrier* (apply related words) 23034

23. S21 OR S22 93104

24. S20 AND S23 **133**

Title Sift 1 - 40

Abstract Sift 2 - 10

Full Paper Sift 3 - 1

**CINAHL (via EBSCOhost):**  **(hits)**

1. health status indicators 60

2. outcome and process assessment 6

3. outcome assessment 385

4. quality of life 23258

5. outcome measure* (apply related words) 23799

6. health outcome* (apply related words) 4404

7. measure* (apply related words) 150238

8. assess* (apply related words) 152356

9. score* OR scoring 59642

10. index 31861

11. indices 94439

12. scale* (apply related words) 46252

13. monitor* (apply related words) 29221

14. S7 OR S8 OR S9 OR S10 OR S11 OR S12 OR S13 306003

15. outcome* (apply related words) 108021

16. S14 AND S15 67549

17. S1 OR S2 OR S3 OR S4 OR S5 OR S6 48520

18. S16 OR S17 87245

19. routine* (apply related words) 18912

20. S18 AND S19 3116

21. facilitat* (apply related words) 24061

22. barrier* (apply related words) 16556

23. S21 OR S22 38731

24. S20 AND S23 **209**

Title Sift 1 - 31

Abstract Sift 2 - 14

Full Paper Sift 3 - 6

**Totals for all three searches:**

**Overall hits - 960**

**Title sift - 152 (808 removed)**

**Abstract sift - 54 (98 removed)**

**Full paper sift - 12 (42 removed)**

**Duplicates - 5**

**Found via Reference Scrutiny - 8**

**Total included in Review - 15**
