# Supplementary material for: The barriers and facilitators to routine outcome measurement by allied health professionals in practice: a systematic review
Source: BMC Health Serv Res. 2012 May 22;12:96. doi: 10.1186/1472-6963-12-96 (PMC3358245; doi:10.1186/1472-6963-12-96)
Supplement: Additional file 2 — List of studies excluded from the review following full paper appraisal stage (N = 42). A table containing the studies excluded from the current review following full paper appraisal. [file 1472-6963-12-96-S2.doc]

The Barriers and Facilitators to Routine Outcome Measurement by Allied Health Professionals: A Systematic Review.

Additional File 2: List of studies excluded from the review following full paper appraisal stage (N=42).

| Sourced From | Article |
| --- | --- |
| MEDLINE | Belazi, D., et al. (2002). Measuring health-related quality of life in the clinical setting. Expert Review Pharmacoeconomics Outcomes Research, 2(2), 109-117. |
| MEDLINE | Bowman, J., et al. (2009). Development and psychometric testing of the Clinician Readiness for Measuring Outcomes Scale. Journal of Evaluation in Clinical Practice, 15, 76-84. |
| PsychINFO | Cape, J. (2000). Clinical effectiveness in the UK: Definitions, history and policy trends. Journal of Mental Health, 9(3), 237-246, |
| CINAHL | Dobrzykowski, E.A. (1997). The methodology of outcomes measurement. Journal of Rehabilitation Outcomes Measurement, 1, 8-17. |
| PsychINFO | Eisen, S. V. (2000). Clinical status: Charting for outcomes in behavioural health. Psychiatric Clinics of North America, 23(2), 347-361. |
| PsychINFO | Erharter, A., et al. (2010). Implementation of computer-based quality-of-life monitoring in brain tumor outpatients in routine clinical practice. Journal of Pain and Symptom Management, 39(2), 219-229. |
| CINAHL | Erharter, A., et al. (2010). Implementation of computer based quality of life monitoring in brain tumor outpatients in routine clinical practice. Journal of Pain and Symptom Management, 39(2), 219-229. |
| MEDLINE | Erharter, A., et al. (2010). Implementation of computer-based quality-of-life monitoring in brain tumor outpatients in routine clinical practice. Journal of Pain and Symptom Management, 39(2), 219-229. |
| MEDLINE | Evans, D. J. (1999). Goal attainment scaling in a geriatric day hospital. Canadian family Physician, 45, 954-960. |
| MEDLINE | Ganz, P. A. (1995). Impact of quality of life outcomes on clinical practice. Oncology, 9(11), 1-9. |
| MEDLINE | Gilbody, S. M., et al. (2002). Routine administration of Health Related Quality of Life (HRQoL) and needs assessment instruments to improve psychological outcome – a systematic review. Psychological Medicine, 32, 1345-1356. |
| CINAHL | Giesinger, J., et al. (2009). Towards the implementation of quality of life monitoring in daily clinical routine: Methodological issues and clinical implication. Breast Care (Basel), 4(3), 148-154. |
| MEDLINE | Greenfield, S. & Nelson, E. C. (1992). Recent developments and future issues in the use of health status assessment measures in clinical settings. Medical care, 30(5), MS23-MS41. |
| MEDLINE | Greenhalgh, J. (2009). The applications of PROs in clinical practice: What are they, how do they work, and why? Quality of Life Research, 18, 115-123. |
| PsychINFO | Greenhalgh, J. (2009). The applications of PROs in clinical practice: What are they, how do they work, and why? Quality of Life Research, 18, 115-123. |
| MEDLINE | Greenhalgh, J. & Meadows, K. (1999). The effectiveness of the use of patient-based measures of health in routine practice in improving the process and outcomes of patient care: A literature review. Journal of Evaluation in Clinical Practice, 5(4), 401-416. |
| MEDLINE | Haywood, K. L., et al. (2009). Continence specialists use of quality of life information in routine practice: A national survey of practitioners. Quality of Life Research, 18, 423-433. |
| MEDLINE | Haywood, K. L., et al. (2009). Continence specialists use of quality of life information in routine practice: A national survey of practitioners. Quality of Life Research, 18, 423-433. |
| MEDLINE | Jacobsen, P. B. (2002). Assessing Quality of Life in Research and Clinical Practice. 16(9), 133-138. |
| MEDLINE | Kazis, L. E., et al. (1990). Health status reports in the care of patients with rheumatoid arthritis. Journal of Clinical Epidemiology, 43(11), 1243-1253. |
| MEDLINE | Lohr, K. N. (1992). Applications of health assessment measures in clinical practice. Overview of the Third Conference on Advances in Health Status Assessment. Medical Care, 30(5), MS1-MS14. |
| CINAHL | Nelson, E. C. & Berwick, D. M. (1989). The measurement of health status in clinical practice. Medical Care, 27, S77-S90. |
| MEDLINE | Pagliari, C., et al. (2005). Adoption and perception of electronic clinical communications in Scotland. Informatics in Primary Care, 13, 97-104. |
| MEDLINE | Phungrassami, T., et al. (2004). Quality of life assessment in radiotherapy patients by WHOQOL-BREF-THAI: A feasibility study. Journal of the Medical Association of Thailand, 87(12), 1459-1465. |
| CINAHL | Pollock, A. S., et al. (2000). Barriers to achieving evidence based stroke rehabilitation. Clinical Rehabilitation, 14, 611-617. |
| PsychINFO | Prabhu, R. & Browne, O. (2008). The use of the Health of the Nation Outcome Scale in an outreach rehabilitation program. Australasian Psychiatry, 16(3), 195-199. |
| MEDLINE | Rogausch, A., et al. (2009). Feasibility and acceptance of electronic quality of life assessment in general practice: An implementation study. Health and Quality of Life Outcomes, 7, doi:10.1186/1477-7525-7-51 |
| PsychINFO | Rock, D., et al. (2001). Issues associated with the implementation of routine outcome measures in public mental health services. Australasian Psychiatry, 9(1), 43-46. |
| CINAHL | Rose, R. L. & Bucknail, T. (2004). Staff perceptions on the use of a sedation protocol in the intensive care setting. Australian Critical Care, 17(4), 151-159. |
| MEDLINE | Silvia, K. A., et al. (2006). Decision aids in routine practice: Lessons from the breast cancer initiative. Health expectations, 9, 255-264. |
| CINAHL | Silvia, K. A., et al. (2008). Implementing breast cancer decision aids in community sites: barriers and resources. Health Expectations, 11, 46-53. |
| PsychINFO | Silvia, K. A., et al. (2008). Implementing breast cancer decision aids in community sites: barriers and resources. Health Expectations, 11, 46-53. |
| PsychINFO | Slade, M. (2002). The use of patient-level outcomes to inform treatment. Epidemiologia e Psychiatria Sociale, 11(1), 20-27. |
| MEDLINE | Slade, M. (2002). The use of patient-level outcomes to inform treatment. Epidemiologia e Psychiatria Sociale, 11(1), 20-27. |
| PsychINFO | Slade, M., et al. (1999). The feasibility of routine outcome measures in mental health. Social Psychiatry and Edidemiology, 34, 243-249. |
| MEDLINE | Slade, M., et al. (1999). The feasibility of routine outcome measures in mental health. Social Psychiatry and Edidemiology, 34, 243-249. |
| MEDLINE | Solari, A. (2005). Role of health-related quality of life measures in the routine care of people with multiple sclerosis. Health and Quality of Life Outcomes, 3(16), doi:10.1186/1477-7525-3-16 |
| MEDLINE | Stamm, T. A., et al. (2007). The use of databases for quality assessment in rheumatoid arthritis. Clinical and Experimental Rheumatology, 25(Suppl.47, S82-S-85. |
| MEDLINE | Thewes, B., et al. (2009). Does routine psychological screening of newly diagnosed rural cancer patients lead to better patient outcomes? Results of a pilot study. Australian Journal of Rural Health, 17, 298-304. |
| CINAHL | Turner-Stokes, L. & Turner-Stokes, T. (1997). The use of standardised outcome measures in rehabilitation centres in the UK. Clinical Rehabilitation, 11, 306-313. |
| MEDLINE | Velikova, G., et al. (1999). Automated collection of quality of life data: A comparison of paper and computer touch-screen questionnaires. Journal of Clinical Oncology, 17, 998-1007. |
| MEDLINE | Vetter, T. R. (2007). A primer on health-related quality of life in chronic pain medicine. Anesthesia & Analgesia, 104, 703-718. |
