# Supplementary material for: The barriers and facilitators to routine outcome measurement by allied health professionals in practice: a systematic review
Source: BMC Health Serv Res. 2012 May 22;12:96. doi: 10.1186/1472-6963-12-96 (PMC3358245; doi:10.1186/1472-6963-12-96)
Supplement: Additional file 3 — Summary of quality appraisal information for included studies. A table containing the quality appraisal and study details for the 15 papers included in the current review. [file 1472-6963-12-96-S3.doc]

The Barriers and Facilitators to Routine Outcome Measurement by Allied Health Professionals: A Systematic Review.

| Additional File 3: Summary of quality appraisal information for included studies. | | | | | | |
| --- | --- | --- | --- | --- | --- | --- |
| **Study Information** | | | **Quality Appraisal** | | | |
| **Author, Date, Country** | **Professional Group(s), N** | **Study Design** | **Sampling Method** | **Description of Data Collection** | **Description of Data Analysis** | **Data Quality** |
| Kay et al. 2001. Canada. | Physiotherapists, N=91 | Quantitative: Questionnaire | Convenience | ++ | + | Simple |
| Jette et al. 2009. USA. | Physiotherapists, N=498 | Quantitative: Questionnaire | Random | ++ | ++ | Simple |
| Van Peppen et al. 2008.  Netherlands. | Physiotherapists, N=167 | Quantitative: Questionnaire | Random | ++ | ++ | Allows complexity |
| Abrams et al. 2006.  Australia. | Physiotherapists  N=318 | Quantitative: Survey | Random | + | + | Simple |
| Chesson et al. 1996.  Scotland. | Physiotherapists; Occupational Therapists  N=184 | Quantitative: Survey | Purposeful | + | ++ | Simple |
| Stokes & O’Neill. 2008.  Ireland. | Physiotherapists, N=15 | Quantitative: Questionnaire | Purposeful | + | - | Different sources used |
| Huijbregts et al. 2002.  Canada. | Physiotherapists, N=42 | Qualitative: Focus groups | Purposeful | ++ | + | Simple |
| Maher & Williams 2005.  New Zealand. | Physiotherapists, N=18 | Qualitative: Telephone survey | Theoretical | - | + | Simple |
| Deutscher et al. 2008.  Israel. | Physiotherapists, N=114 | Mixed Methods:  Qualitative- Focus Groups; Quantitative: not relevant to the present review, i.e., did not investigate facilitators and barriers to routine outcome measurement | Purposeful | ++ | - | Simple |
| Copeland et al. 2008.  New Zealand. | Physiotherapists  N=381 | Mixed Methods:  Qualitative- Focus groups.  Quantitative- Questionnaire | Purposeful | ++ | + | Allows complexity |
| Russek et al. 1997.  USA. | Physiotherapists;  Occupational Therapists, N=102 | Quantitative: Survey | Purposeful | ++ | ++ | Simple |
| Blenkiron 2005. UK. | Occupational Therapists, N=118 | Mixed Methods: Survey | Purposeful | ++ | + | Simple |
| Colquhoun et al. 2010.  Canada. | Occupational Therapists,  N=3 | Qualitative: Survey | Purposeful | + | ++ | Simple |
| Skeat & Perry 2008.  Australia. | Speech and Language Pathologists; Occupational Therapists  N=16 | Qualitative: Open ended interviews | Theoretical, purposeful | ++ | ++ | Allows complexity |
| Simmons-Mackie et al. 2005. USA & Canada. | Speech and Language Therapists, N=94 | Mixed Methods: Survey (online) | Purposeful | ++ | + | Simple |
| Key for description of data analysis and data collection:  **++ Good:** Description is clear and contains sufficient detail allowing ease of precise replication.  **+ Some:** Description is clear but further detail would be required to aid clarity/allow precise replication.  **- Partly**: Insufficient description of method; precise replication based on published information would not be possible. | | | | | | |
| Key for Data Quality (based on Kolehmainen *et al.*^[19]^):  **Simple:** Data is mainly descriptive (whether collected qualitatively via structured means or via quantitative questionnaires).  **Allows Complexity:** Data is rich enough to allow deeper analysis than description/frequency information, or allows the comparison between groups of different participants.  **Different Sources used**: Data collected at more than one time-point, or via multiple standardised questionnaires. | | | | | | |
